# “But my horse is well cared for”: A qualitative exploration of cognitive dissonance and enculturation in equestrian attitudes toward performance horses and their welfare

**DOI:** 10.1017/awf.2025.10028

**Published:** 2025-07-24

**Authors:** Erica Cheung, Daniel Mills, Beth Ann Ventura

**Affiliations:** Animal Behaviour, Cognition, and Welfare Group, Department of Life Sciences, Joseph Banks Building, Green Lane, Lincoln LN6 7TS, UK

**Keywords:** Animal welfare, care ethics, cognitive dissonance, enculturation, horse welfare, performance horses

## Abstract

There is concern amongst the public, equestrians, animal welfare organisations, and horse-sport governing bodies regarding the welfare of performance horses, but equestrian culture appears slow to change. The present study seeks to increase our understanding of human factors underlying the persistence of welfare-compromising management and training practices within the performance horse world. Individual, semi-structured interviews focused on equestrians’ attitudes were conducted with 22 equestrians from classical equestrian disciplines in the US, Canada, and the UK. Interview transcripts were analysed using reflexive thematic analysis. Five main themes were identified: perception of welfare issues; conflicting conceptions of a good life; objectification of the horse; instrumentalisation of horse care; and enculturation. Participants perceived and were concerned about horse welfare, but expressed dissonance-reducing strategies, including trivialisation, reframing and justification. Participants shared conflicting conceptions of a good life and described how equestrian activities may infringe upon horse welfare. Objectification of horses was among the attitudinal factors identified that may permit persistence of harmful practices, while the instrumentalisation of care theme showed how management practices often focused on performance and the horse’s job more than care *about* the horse. Finally, enculturation (the process of adopting attitudes and behaviours of a culture) in equestrianism may be fundamental to maintaining practices and attitudes that compromise horse welfare. These findings provide an enhanced understanding of why horse welfare issues persist in classical equestrian disciplines and may inform future human behaviour change strategies to promote improved horse welfare.

## Introduction

Performance horse welfare is the subject of increased attention from the public, animal welfare organisations, horse sport governing bodies, researchers and equestrians themselves (Waran & Visser 2022; Heleski *et al.* 2023; Fiedler *et al.* 2025). Although performance horses may be extremely well cared for in terms of their physiological needs, they are often subjected to aversive training methods that cause fear and pain, restrictive housing and turnout, and limited to no social contact with conspecifics (Mills & Nankervis 2013; Horseman *et al.* 2017; Dubois *et al.* 2018a,b; McGreevy *et al.* 2018). While there is significant evidence of the welfare effects of common equine husbandry and training practices (McGreevy *et al.* 2018), translation of research into effective change in practice remains challenging (Pickering & Hockenhull 2019; Reidpath *et al.* 2022). Expanding research focus toward human motivations in the context of horse management and training practices has been proposed as a way forward to improve horse welfare (Furtado & Rendle 2022; Wolframm *et al.* 2023).

To address welfare concerns specific to performance horses, the International Federation for Equestrian Sports (FEI) recruited an independent group of welfare scientists and other experts in 2022 to form the Equine Ethics and Wellbeing Commission. Among the outcomes of this commission was the ‘Be a Guardian’ initiative to promote and improve welfare of performance horses (Waran & Visser 2022). FEI president Ingmar De Vos explained guardianship as “…*meeting the horse’s natural needs – such as sufficient eating time, social interaction, and exercise*…” (FEI 2024). De Vos goes on to say that guardianship focuses on *“what is best for the horses, recognising them as unique and valuable beings deserving of human protection, trust, and respect. This evolution redefines the human role as that of a ’caretaker,’ with a responsibility to ensure a good life for horses*” (FEI 2024). These statements suggest that the profession broadly acknowledges the need to protect and improve performance horse welfare across several domains. Furthermore, it highlights the fundamental role of humans in caring *for* horses and *about* horses’ well-being, and the need for human behaviour to change to fulfill this responsibility.

The Five Domains model is a conception of animal welfare built around the ethos of providing a ‘good life’; in the case of performance horses, it has been proposed as a guide for the education of equestrians in the evaluation of horse welfare (Waran & Visser 2022). This model suggests that welfare is comprised of the domains of nutrition, environment, health, behavioural interaction, and mental state; it therefore also implies caring *about* animals as subjective beings through the mental state domain (Mellor *et al.* 2020; Mellor & Beausoleil 2020). Application of the Five Domains model requires cognitive and behavioural action in order to care *about* and *for* horses. Further, according to the care ethics framework, the attitudes, emotions, and values that individuals hold contribute to *caring about* the needs of another being, which in turn motivate the behaviours that comprise *caring for* an individual (Tronto 1993; Held 1995; Noddings 2013). Attitudes are the relatively consistent evaluation of any given subject or object (Shavitt 1990; Stangor 2020). They not only demonstrate what individuals may think and feel, they are also predictive of behaviour (Glasman & Albarracín 2006) and are strengthened by performance of those behaviours (Davidson & Jaccard 1979; Kraus 1995). The attitudes that individuals hold are shaped by emotions, experiences, and socio-cultural norms and can therefore be contextual (Jhangiani & Tarry 2022). For example, one might hold an attitude towards horses in the context of owning them as companions that is different from the attitude held towards horses in equestrian sports. From this, it can be seen that culture is a potentially important influence on attitudes. A culture can be defined as the collection of social behaviours, institutions, norms, knowledge, beliefs, customs, capabilities and habits of individuals in a group (Tylor 1871). This fits the concept of equestrianism where practices, norms, knowledge and customs are certainly passed down through generations. A good example is the maintenance of the fundamentals of horse training over the centuries (Waran *et al.* 2007).

Although previous studies have worked to identify barriers to changing practices that compromise horse welfare (Furtado *et al.* 2021; Luke *et al.* 2023, 2024; Mauricio *et al*. 2024; Fiedler *et al.* 2025; Ross *et al.* 2025), there is a lack of information on the underpinnings of equestrians’ attitudes within the performance horse industry that may fundamentally drive their practices. Understanding those human factors that impact negatively on horse welfare is essential to promote positive change for this welfare (Furtado & Rendle 2022). Therefore, the present qualitative study aims to explore the attitudes and associated elements that may influence how equestrians care *for* and *about* performance horses. The study was anchored in the aim to explore the attitudes of equestrians towards horses and horse sports in general as well as their specific perspectives on performance horse welfare. We were also interested in their conception of what a good life for horses looks like for all horses but with an emphasis on performance horses. Finally, we examined the potential influence of the public’s input as an external factor potentially affecting how performance horses are managed and trained.

## Materials and methods

### Positionality of the research team

Researchers play a critical role in the development of meaning of qualitative data (Creswell & Poth 2016; Braun & Clark 2022). This is a strength in qualitative research that contributes to discovery when addressing the complexity of human attitudes, perceptions and thoughts. To ensure the methodological rigour of qualitative research, transparency relating to the potential influences of researchers is essential. EC comes to this research with a post-graduate degree in animal behaviour and welfare and maintains a personal goal of contributing to improving animal welfare. EC also owns horses and has been involved in horse training and sports for several years. BV holds an academic appointment as an animal welfare scientist. She has held positions in both North America and the UK and often employs mixed method approaches to address how the perceptions of key groups may inform resolutions to animal welfare challenges. She has long enjoyed working with horses in a variety of contexts but is not part of the performance horse sector in any capacity. DM is a professor of Veterinary Behavioural Medicine, with a PhD in equine behaviour and management. He uses a wide range of quantitative and qualitative work in his research. He is a recognised specialist in veterinary behavioural medicine, including horses. Within these roles, we were committed to honouring the diverse perspectives of our participants and interrogating the evidence with open minds.

No formal theoretical framework was used to frame this study, but we took an interpretivist approach (Creswell & Poth 2016; Pulla & Carter 2018; Alharahsheh & Pius 2020) to the current research process as this paradigm allows researchers to gain insight into human experiences and culture by interpreting meaning from what participants contribute. The interpretivist approach holds the ontology that there can be multiple perceived realities constructed by our own individual experiences and social interactions, the epistemology that the representation of these realities is co-constructed by the researcher and the research participants, and the axiology that individual values must be honoured but are also influenced by each other. Effort was consciously given to listen to participants without judgement and respect their experiences, thoughts, and opinions as their own realities.

### Ethical considerations

The study was reviewed and granted ethical approval by the University of Lincoln Ethics Committee (ethics reference UofL2024_16292). Participants were provided with a study information sheet and an opportunity to ask questions about the research before signing a consent form. Prior to the interview, participants were reminded that the interview would be recorded and that they could withdraw consent until the point when their data were anonymised. Participants were assured that sharing their thoughts, perceptions, and observations regarding animal welfare was voluntary and their participation was confidential. A debrief was provided at the end of the interview with another reminder that the participant could withdraw consent up until data anonymisation.

### Recruitment

Participants were recruited through a purposive and convenience sampling approach between March 26 and June 17, 2024. An advertisement and invitation to participate was shared to equestrian interest groups across social media platforms, including Facebook, Instagram, X, and LinkedIn.

Inclusion criteria for the study were equestrians (aged 18 years or older) involved at any level in classical equestrian sports, such as dressage, showjumping, hunters, and eventing. Equestrians were defined as anyone involved with performance horses, including coaches, horse trainers, stable owners/managers, horse owners, grooms, riders, and competition judges. Exclusion criteria consisted of colleagues, friends, or family of the researchers involved in the work, and equestrians outside of classical disciplines or who did not have an involvement in equine sports.

### Participants

A total of 27 individuals were initially recruited; however, two chose to withdraw their data due to concerns about sharing sensitive information, two elected not to proceed with the interview process due to time constraints, and one failed to attend their interview. This left us with 22 equestrians who participated (Table 1). Participants included three individuals identifying as male and 19 as female. They resided in the United States (n = 10), Canada (n = 7) and the UK (n = 5) at the time of the study. They were primarily involved in the horse industry as hunter/jumper riders and coaches, dressage riders and coaches, grooms, stable/livery owners or managers, and eventers.

**Table 1.** Demographic characteristics of participating equestrians (n = 22) involved in a qualitative study on performance horse welfare

Category	N
**Discipline** *	
Breed showing	1
Dressage	5
Eventing	3
Equitation	1
Hunters	6
Saddleseat	1
Showjumping	8
**Participant role(s) within industry** *	
Breeder	1
Coach/trainer	4
Groom	2
Livery/stable owner	3
Livery/stable manager	4
Competitor	20
**Country of residence**	
Canada	7
United Kingdom	5
United States	10
**Gender**	
Woman	19
Man	3
**Age**	
18–24	3
25–44	15
45–60	4

* Numbers do not always add to 22 as participants could hold multiple roles and/or participate in several disciplines

### Data collection

Semi-structured, one-on-one interviews were used. An interview guide was developed based on the aims of the research, piloted amongst five individuals (contacts of EC ineligible to participate in the study), and questions refined for clarity. The final guide consisted of eight primary open-ended questions aimed at determining attitudes towards horses and horse sports, thoughts about a good life for horses, attitudes and perceptions of welfare issues, and attitudes towards the public’s views of horse welfare in sports. Each question had multiple probes, which were modified as needed based on participants’ responses during the interviews (see Interview Guide Protocol in online supplementary material). Interviews were conducted between April 1 and June 17, 2024 via Zoom (version 5.17.11). Holding interviews virtually allowed inclusion of a diverse study population that would not otherwise have been feasible (Greenspan *et al.* 2021). Throughout the data collection period, patterns in participant responses were noted. Sufficient information power of the study (Fusch & Ness 2015; Braun & Clark 2021) was considered when there did not appear to be novel insights provided, which occurred between the 19th and 22nd interview. We aimed to include a breadth of equestrian disciplines and participant roles within the horse performance industry. Interviews were recorded with the written and verbal consent of participants for later transcription. The recordings were reviewed for familiarisation then transcribed manually by EC. Transcripts were then reviewed twice by EC for accuracy of transcription and any personally identifying information was removed to maintain participant anonymity. Interview recordings were permanently deleted, and the data were then considered anonymised.

### Data analysis

The six-phase approach by Braun and Clarke (2006, 2022) was used as a framework for the reflexive thematic analysis undertaken for this study. Interview transcripts were reviewed for familiarisation, interviewee quotes highlighted, and reflective memos were used to note initial interpretations of the data. A deductive approach was first taken in the coding of the data to meet the aims of the research; for example, coding for a participant’s perception of welfare issues where we sought to identify this through questions in the interview. An inductive open coding method was then used to generate descriptive codes driven by the rich data. EC led this process, applying analysis at the level of each transcript first and then examining across all transcripts to understand commonalities and divergences in themes and subthemes as these were developed. Codes were reviewed in critical discussion with DM and BV and further defined.

## Results and Discussion

**Figure 1.**
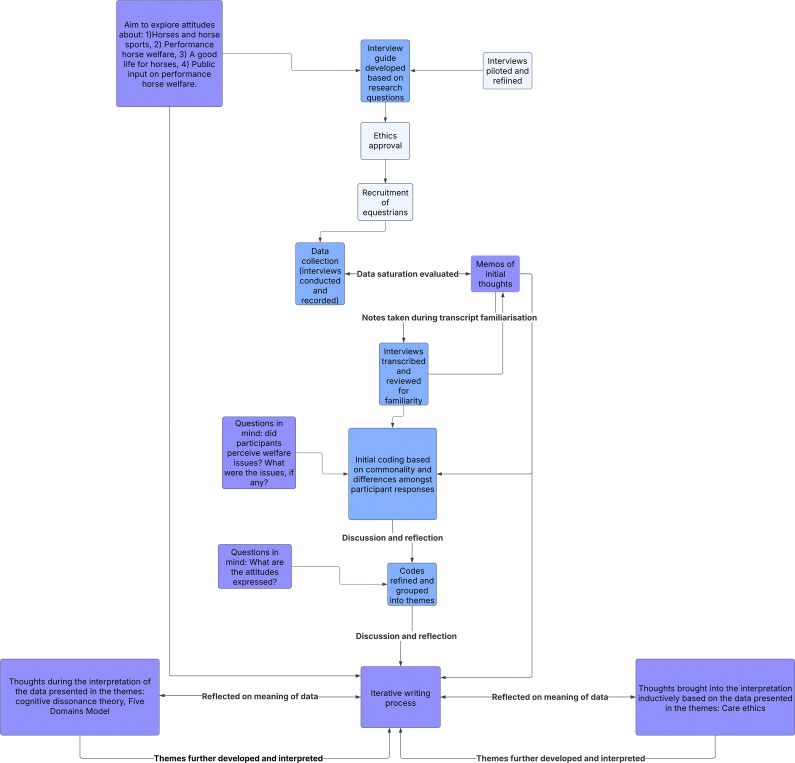
The research process for the qualitative study using reflexive thematic analysis to explore equestrians’ (n = 22) attitudes and perceptions around performance horses and their welfare. The stages of the research process from development to the iterative writing process are shown.

Themes were constructed by identifying patterns across the data and reflecting upon the meaning and connections between them. They were further refined through the writing process. Interpretations of themes with respect to conceptions of welfare and the good life of a horse were framed within a Five Domains perspective (Mellor *et al.* 2020). As themes related to attitudes toward horses, welfare, and other topics were interpreted, what it means to care for another being based on care ethics (Held 1995; Noddings 2013) was brought into the iterative process. The role of cognitive dissonance theory (Festinger 1957) also came into the interpretation as we reflected on explanation for some of the findings. These theories helped to make sense of participants’ contributions as the deeper meaning of the themes was formed. Figure 1 depicts the research process from beginning to end. Quotes from participants are used in reporting of the results to illustrate the findings.

**Figure 2.**
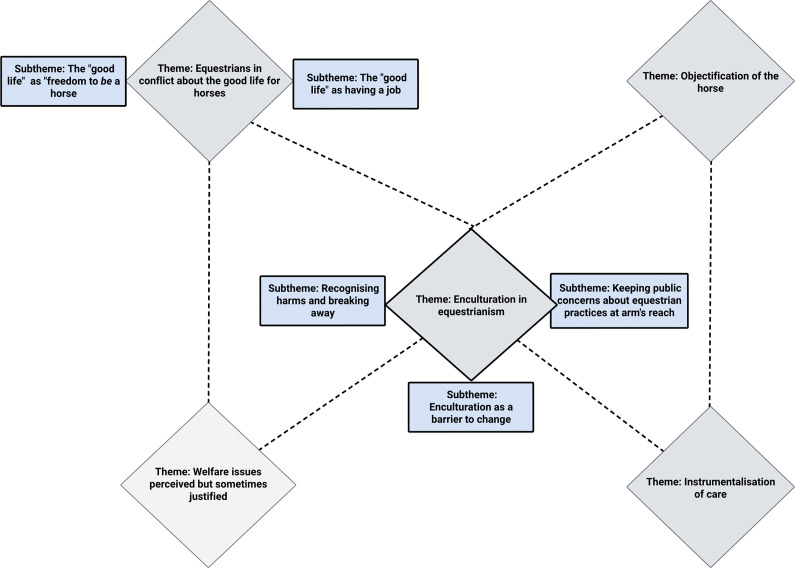
The research process for the qualitative study using reflexive thematic analysis to explore equestrians’ (n = 22) attitudes and perceptions around performance horses and their welfare. The stages of the research process from development to the iterative writing process are shown.

Five overarching themes were developed. Theme 1 describes participants’ attitudes toward horses and perception of performance horse welfare, while Theme 2 highlights the conflict experienced in their conception of a good life for horses. Themes 3 and 4 show how performance horses might be objectified and how the instrumentalisation of care subsequently occurs. Finally, Theme 5 explores the process of enculturation and the possibility for equestrians to break away from traditional attitudes and practices. Figure 2 depicts proposed relationships between the themes discussed.

### Theme 1: “But my horse is well cared for”: Welfare issues perceived but sometimes justified

To care *about* horse welfare, equestrians must first demonstrate awareness of welfare issues. In responses to prompts to share their observations and thoughts on performance horse welfare, many participants perceived welfare issues in the equine industry and also often spontaneously raised concerns without prompting when discussing common training and management practices. Participants commonly reported welfare issues within all aspects of equine care and training, including whip use, harsh training equipment, physical punishment, drugging horses, lack of turnout and lack of social contact. For example, with respect to training and showing practices: “*I’ve seen illegal bit use”*, said P3, *“I know personally of multitude of trainers who drug the horses. They drug horses for lessons, they drug them for horse shows”.* P22 commented, *“I see horses getting lunged into the ground”*, while P6 stated*, “And I’ve watched coaches get on the horse and then get it to stand still and then beat the crap out of it”.* Similarly, P8 shared,

*“There was one woman last year at a show that had her horse tied up to the side of the lorry and he was being a bit naughty, he was a bit excited so she tied him up to the side of the lorry with a rope from quite high so he couldn’t move his head and just kept smacking his legs with a whip until he stopped moving them. Every time he moved them she smacked him”.*

While they perceived welfare issues and expressed concern for the well-being of horses, participants also justified practices they acknowledged as harmful by employing several strategies, including reframing and trivialisation. Reframing issues allows individuals to change their conceptual viewpoint and see a negative scenario in a different, and often more positive, light (Throop & Castellucci 2008; Robson & Troutman-Jordan 2014). An example of a welfare concern that was often positively reframed by participants is the permanent keeping of show horses in stalls, except for hand walks or ridden activity, e.g. *“I’ve seen a lot of show horses that are very busy at shows that go for lots of walks. And, you know, maybe they do spend 22 hours a day in a stall when they’re at horse shows, but they seem fairly content”* (P22). Similarly, P3 discussed the benefits of turnout and then justified not providing it in some cases: *“It’s good for their mental health as well as getting their zoomies out. Except the two naughty mares who refuse to be caught so they lost their privileges of going out at all. But they’re happy”.*

Another form of justification is trivialisation (Festinger 1957; Simon *et al.* 1995), wherein people reduce the cognitive importance of an issue by minimising it. This appeared to be employed by some participants in their minimisation of situations that may lead to compromised welfare. For example, when asked if they observed any concerns within their own or other equine sports, P10 responded, *“For the most part, not really…I see some stressed horses. So I don’t see horrifying things happen to horses”.* Another spoke of harsh bits and horses ridden behind the vertical, a practice believed to cause discomfort as the horse’s head is pulled in close to their chest (see König von Borstel *et al.* 2024): “*Which maybe isn’t particularly concerning”*, they quickly added, *“but, in my opinion, isn’t conducive to the horse… even I’ve seen bloody mouths in person and I have seen spur marks that were bloody and big in person”* (P1). In both examples, participant responses suggested that certain issues were trivial in comparison to others.

Trivialisation was also observed in participants’ discussions regarding lack of turnout, which many expressed concern about while simultaneously diminishing these as welfare concerns. P10 reflected on the day-to-day management of their horse and others, “*We* [equestrians] *put them* [horses] *in this box however many hours a day, like, you know they’re out for four hours a day. That’s still 20 hours a day in a box, right? And that kills me sometimes. But my horse is well cared for”.* This exemplified how some expressed concern for horse welfare but then trivialised their issues of concern as their horse was generally well cared for.

It is not suggested that participants believed that welfare-compromising practices are acceptable. However, participants appeared to not only perceive and demonstrate genuine concern about welfare issues but also, in some cases, justified, reframed and trivialised these very same concerns. This suggests that there is a source of dissonance and participants must resolve the conflict between their beliefs and acceptable practice (Festinger 1957). Justification methods, such as reframing and trivialisation, serve the function of dissonance reduction (Dhont 2019). The attitudes that further contribute to potential dissonance amongst equestrians are next to be investigated.

### Theme 2: “Just let them be a horse”: Equestrians in conflict about ‘the good life’ for horses

How equestrians conceive of a good life for horses is important in understanding how they may address horse welfare issues. *Theme 2* highlights the potential internal conflict that equestrians may confront when conceptualising a good life for performance horses. This form of conflicted perspective has been discussed within the context of dressage (Ross *et al.* 2025) but is found across disciplines in the present study.

#### Subtheme 2.1: The good life as having a job

Nearly all study participants identified having a job and enjoying it as an important part of a good life for a horse. For example, P9 stated:

*“… a good life for a horse is one where they have a job that suits them. …I’ve known enough horses that…were big sport horses…and they kept trucking and trucking and the moment that somebody decided that they needed to retire because they just couldn’t do it anymore, they dropped dead … I think they’re the most happy … when they have a job that suits them and…maintained to manage that job”.*

P3 similarly suggested that having a job is indeed necessary for a horse’s well-being: *“I think the public sees our horses that they should be more pets rather than have a job and they* [the public] *don’t understand how even for their bodies they* [horses] *need to work and do things. If they don’t they would actually not thrive”.*

As seen by these examples, the horse’s job was considered by most participants as fulfilling to the horse’s mental and physical well-being. However, several also identified how the horse’s job to some extent restricts their care. This was discussed as a trade-off where turnout or other management factors are adjusted to ensure prioritisation of the horse’s job. This suggests that participants were aware of the detriments to horse welfare imposed by their performance activities: *“A good life for a horse, you know, has its caveats just like anything*”, said P15. An example of the imposition on the horse was described by P18, *“Yeah, I mean the ideal from a living situation is as much turnout as possible within the confines of whatever is their use”.* Restricting turnout and access to other horses for the purpose of preventing injury was the reasoning most often discussed by participants. Less frequently, some mentioned the set-up of existing housing infrastructure.

P4 justified the potential compromises made in this situation as follows:

*“Like ideally, if they could just live in a field and be with other horses, that would be great. But that’s not the way the industry works. We can’t do that. That’s not how it goes. So I try to make sure, for me, horses need to have access to good hay, good water. They need to be able to socialise with other horses. Turnout together, not always an option, but at least sniffing noses and some little grooming sessions between each other while they’re being held; that’s what we do. And even hand-grazing together will take two out at a time or whatever. So it’s kind of like they’re in a herd, but they’re safe. Not kicking* [the] *crap out of each other. So happy horses to me. Happy life”.*

#### Subtheme 2: The good life as freedom to ‘be a horse’

Most participants also spoke about a good life, or other things they wished all performance horses could have, in terms of the horse being able to just be themselves as a horse. For example, P1 summed up what they would want: *“…yeah, like just let them be a horse is a big one for me”.* Likewise, P15 said, *“I guess a happy horse would be one that is kind of encouraged to be as much of a horse as possible, like natural”.* These comments suggest that participants consider that good welfare consists of behavioural freedom and autonomy in addition to basic physiological needs being met.

Importantly, when participants were asked if horses in their sport experienced a good life in accordance with their descriptions, most were, at best, cautiously optimistic. For example, P16 responded,

*“In terms of taking care of a beast so that it is well-fed and useful for its task, yes. But in terms of honouring the horse as a horse and letting them be herd animals, letting them have turnout, letting them eat as their bodies are naturally meant to eat, no, absolutely not. We* [equestrians] *are not* [providing horses a good life]*”.*

Similarly, P22 articulated their experience as a conflict related to caring *about* the horse when caring *for* them, saying,

*“It’s really hard to sometimes parse out what is best for who sometimes. There’s a lot of people making decisions that are best for them, but not in the best interest of their children. And I think it’s very similar with horses. So I do think our whole sport kind of needs to move in a direction where we have the welfare of the horses more in mind”.*

In summary, participants spoke about horses as subjective individual beings in that they wanted horses to *be horses*, and that allowing this would enable horses to be happy and experience a good quality of life. However, this contrasted with the common view expressed that horses also need a job, whilst accepting that the performance aspect often places restrictions on horse management. Indeed, many expressed concerns that performance horses do not experience a good life in terms of *being a horse.* This suggests that participants may be conflicted as they consider how to balance considerations of what a good life for a horse consists of with their focus on horses’ function in sport. This potential source of dissonance might influence the horse-human relationship and how participants make horse care-related decisions.

### Theme 3: “They need to do their job”: Objectification of the horse

*Theme 3* explores an aspect of participants’ attitudes towards performance horses and how they are valued, offering an insight into the horse-human relationship in this context. In this theme, objectification of horses was interpreted based on Nussbaum’s (1995) definition, which involves perceiving an individual as instrumental, fungible, violable, and owned while denying autonomy, agency, and subjectivity. Participants admired traits in horses that contribute to their instrumentality (usefulness) and violability (vulnerability to violation), such as perceived willingness and compliance. For example, P14 responded, *“I think just a horse that’s willing to work with you and has a good demeanour is very important”.* Equally clear on the topic was P2, *“I like nice horses. You know, they just do as they’re asked”*, and P6 who simply stated, *“I like horses that are respectful”.* While not all presented the horse’s work as a choice (*“Where there is a job to do and I expect them, you know, to do it to the best of their ability…But in the end, they do have a job to do. So they need to do their job”* [P11]), others implied that horses want to participate in performance activities, *“I think if you look at the horses that are successful at their jobs, regardless of their jobs, it’s that they kind of, they wanna be a part of the team. They wanna do the job, they wanna do the right things, they wanna put the effort in”* (P9). P16 similarly proposed that horses consent to their use:

*“It’s about the temperament. It’s about the heart. Do they want to do this job, or at least are they open to doing this job? What do they want?…You can make horses do anything. But if you want to do a partnership and have a horse for many years that serves you well, as much as you serve them, They have to want to do the job”.*

Participants also mentioned fungibility, or the replaceability, of horses in sport. Some spoke of their own experience, such as P9, who explained choices in selling a horse that was *“least productive”.* They went on to say how the sale of the horse would help support other, more useful, horses. Others discussed their observations of equestrian culture, *“I’ve seen some people get another horse, get a new horse, sell the horse. I’ve seen quite a bit of that”* (P2). One participant explained how infrequently they observed owners at a large livery keeping horses over the long term:

*“It’s not often if they haven’t got a job and they haven’t got a purpose. They’re sent somewhere else, they’re sold, it’s not worth keeping one which I find very sad. They usually, they just go up for sale. Maybe the mares will go as brood mares, the geldings just go for sale as a quiet ride or, and when they go the people don’t really care. There’s no follow-up on them. They’re sold, that’s it. Like I find that really sad that there’s as soon as they can’t do a job, that’s it, they’re gone”* (P8).

Other studies have documented attitudes towards horses as being based on ‘love’ (Mauricio *et al.* 2024; Ross *et al.* 2025). However, the interpretation of participants’ attitudes in their relationship with horses in the present study seems to be focused on an instrumentalisation of the horse in the relationship. What may be most valued about the horse is traits related to the perceived willingness of the horse to be used in equestrian activities. Interestingly, a spectrum of objectification was interpreted, where in some cases horses are considered consenting to their instrumentalisation and, in other cases, horses are expected to serve the purpose set out for them, regardless of their willingness. Objectification may influence other aspects of how equestrians view horses and care about them beyond their immediate relationships. Where this objectification may stem from is expanded on in *Theme 5* and requires further exploration in future research.

### Theme 4: “A happy athlete is going to produce results”: Instrumentalisation of care

Participants emphasised the high level of care that performance horses receive throughout the interviews, especially when asked what they would like the public to know about performance horse welfare. For example, P6 said, *“Like in the upper levels, they’re treated better than humans”.* P3 also related performance horse care to a human care standard, *“Oh, sport horses are cared for better than most people’s children… Like if I die and get to come back as something I want to be a middle-aged woman’s sport horse because I will have the* [best] *care”.* In addition, P13 explained the extent that they believe most equestrians go to ensure the care of their horses:

*“Most people will go without things themselves. They’ll eat beans on toast for a month if it meant their horse was shod, had the physio, saw the dentist, they had their lesson. They do all of that. The majority of people sacrifice for themselves in order to better the welfare of their own horses”.*

Based on how participants highlighted the extent to which they care for their horses as something they wanted the public to know, participants were aware that the care they provide is judged by the public. However, some justified their focus on horse care to indicate that the measures taken to provide good welfare created positive outcomes in terms of usage of the horse, suggesting that such care had become instrumentalised such that it confers tangible outcomes that serve the interests of the caregiver rather than the care recipient (Fowers 2010; Sedgewick 2013; Lehrer *et al.* 2022. P4 and P11 are exemplars of this theme: *“I always strive for happy horses. A happy horse is a happy athlete, and a happy athlete is going to produce results”* (P4) and *“But really thinking about the horse first. And once you do that, the horse will give you everything, right. They will try their hardest for you”* (P11).

The ability to produce results in terms of performance was not the only motivator discussed in relation to providing good welfare. A few participants also broadly framed caring for horses according to the highest welfare standards so as to protect social licence (the degree to which an industry is accepted and trusted by society and its interested groups [Heleski 2023]) to continue using horses in sport. P9 explained the critical nature of horse care in this respect:

*“The people that feed the industry are the ones that have to work on a budget. And if that budget gets blown by bad management or bad facilities or bad situations, the industry is gonna fall apart. So everybody has to feed in to taking care of those details. And I think in general it happens, and I think* [we need to] tell those PETA mother*ers that we’re taking care of business and they should just butt out”.

They went on to caution, *“You have to really carefully navigate the presentation”* (P9), suggesting that care practices are important in addressing concerns around public perception of the industry.

This theme provided evidence of how prioritisation of a horse’s job plays a role in participants’ choices concerning their horse’s care, such that the horse’s care was framed within their function as a sport horse. Others have interpreted this to mean that competition goals are a determinant in horse management and training (Luke *et al.* 2024). While this may be a reasonable conclusion, it is proposed here that this is an outcome of the objectification of the horse, which has led to instrumentalisation of care. It is possible that instrumentalisation of care may be facilitated by equestrians failing to perceive their horse’s needs from an empathic standpoint, meaning they may not consider the horse’s perspective. This is related to the occasional anthropomorphic framing of horse’s needs in *Theme 2*, where participants described having a job as part of the conception of a good life for horses.

### Theme 5: “…People get enculturated into a sport”: Enculturation in equestrianism

*Theme 5* demonstrates how enculturation may occur as individuals enter equestrian culture and adopt the attitudes and practices of the culture. Enculturation is “*the process of learning a culture in all its uniqueness and particularity*” (Mead 1963). For an individual to participate in the culture, they must develop an understanding for, and competency in, the practices and knowledge of the cultural scheme, which is socially distributed (Menary & Gillet 2016. Cultural schemes become appropriated by individuals and internalised (Menary & Gillet 2022), thereby serving as a powerful motivator for beliefs, behaviours, and perceptions, all of which influence how cultural knowledge is evaluated (Spiro 1987 Poole 1994). Enculturation is most powerful during childhood, and occurs almost unconsciously during this period (Herskovits 1949; Shimahara 1970).

When participants were asked how they became involved in equestrian sport(s), all responded that they entered the equestrian world as children, between the ages of 4 to 13 years old. Nearly all expressed an inexplicable and undeniable early attraction as their reason for getting involved with horses. Participants frequently described themselves as *“crazy”, “obsessed”* or *“addicted*”. For example, one participant commented, *“I just was a horse-crazy kid”* (P4), while P12 shared, *“I’m one of those people that got into it* [horse riding] *as a kid and I was always into it. I was just one of those that was born wanting to ride”.*

Regardless of childhood motivations for engaging with horses, participants became equestrians as they participated in equine sports and were exposed to equestrian culture. Here, participants described their progression into equestrian culture from childhood to adulthood by learning from horse trainers and equestrian coaches. This form of intergenerational teaching and learning is a key feature of enculturation (Herskovits 1949; Shimahara 1970). Most participants indicated that trainers and coaches strongly influenced their attitudes towards horses. This may set the tone for how individuals relate to horses in the horse-human relationship. One example is what is proposed here as the first manifestation of objectification of the horse, as youth ‘outgrow’ their first pony or horse and replace them based on changing performance goals typically guided by their coaches. Acceptance of common care and training practices also occurs as a result of early experience under the mentorship of coaches and trainers. For example, one participant reflected on the significance of their trainer’s instruction: *“Whenever you’re young, you don’t really know if anything’s wrong. … If you’re six years old and your trainer’s been teaching for 50 years, you’re going to listen to what she says”* (P14). Another recalled their early experiences:

“…*the horses really were not treated very well. And in retrospect, it really bothers me to think about how they were treated. They were not housed very well. They worked several hours a day. They never got to go outside. It was just not a good set-up. But I was a child and I didn’t know any better…. there was some really unpleasant situations where coaches were, you know, pushing children and telling them to, like, punish the horses”* (P10).

Exposure to these management, training, and attitudinal factors in equestrian culture in childhood appeared to normalise harmful practices for some participants. For example, one individual shared:

*“… The way people get enculturated into a sport, there’s certainly people who have sort of set the tone that dressage horses have to be kept in this box all the time… you get enculturated into it. So if people are saying this is how dressage horses live or this is how hunters live or this is how jumpers, we don’t turn them out ’cause they’re gonna get hurt, you know. You kind of take that as that’s normal…”* (P19).

Normalisation occurs as a result of long-term exposure to practices and attitudes portrayed by a collective, resulting in coherence of these practices and attitudes, cognitive participation by social members, and adaptation to the norms (May & Finch 2009). This contributes to the enculturation process and maintenance of enculturated practices and attitudes.

#### Subtheme 1: Enculturation as a barrier to change

Enculturation leads to the perpetuation of practices and attitudes through maintenance of norms within the culture (Herskovits 1949; Shimahara 1970). In this way, enculturation is a potential barrier to change. For example, P5 reflected: *“…There’s a piece of it that’s militaristic from tradition. So I think when that’s been ingrained in you then you definitely are hesitant to change”.*

In response to questions about welfare priorities in sport and probes about where education and information for equestrians should come from, participants frequently offered suggestions on how performance horse welfare could be improved. These suggestions often focused on the need for a figurehead from within the equestrian industry to promote change: *“…what we need is people within the industry that other people respect to start questioning what we’re doing”*, P5 explained, while another felt “*some sort of celebrity”* from within the culture was needed that held people’s *“respect and can get people’s attention to kind of be the advocate”* (P20).

Once enculturated, participants appear to continue *“looking to horse people from the past who’ve had good success…”* (P4), suggesting that alternative sources of influence have little effect. Selective uptake or overreliance on information from within the cultural bubble, as described by participants, may serve to mitigate exposure to conflicting evidence and ultimately reduce the possibility of experiencing cognitive dissonance (Festinger 1957; Cotton & Hieser 1980; Cotton 1985; Jean Tsang 2019). It potentially also supports the persistence of traditional practices, as P11 explained, “…*it’s really on those people that have been doing it for a while and have proven to be successful that need to lead the next generation”.* In this way, encultured practices and attitudes may carry over from one generation of equestrians to the next.

Another barrier to widespread change within the industry created by enculturation is the potential for rejection from the culture itself when trying to go against the norms (Marques *et al.* 2001). This seemed evident among several younger participants in this study, who brought up hesitancy to speak about horse welfare issues that concerned them, *“You know, you see it, you’re talking about it, but not a lot of people talk about it. I mean, I’ve even thought about like reaching out to* [equestrian sport organisation]*, but I’m also scared I’m going to get banned. So I’m like, I have to be careful because then I can’t show anymore”* (P7).

#### Subtheme 2: Keeping public concerns about equestrian practices at arm’s reach

Resistance to those perceived as outsiders (i.e. non-equestrians) also appeared key to maintaining and deepening enculturation within the *status quo* of equestrian culture in this study. This resistance was sometimes presented as a general scepticism towards public input. For example, P6 expressed doubt regarding the potential role of non-equestrians in promoting performance horse welfare,

*“I think the public will help to a degree but I think it’s going to have more fire under it if it’s the trainers and the barn owners and it’s that the people with some stature in the horse industry if they speak up. Because the public* [is] *kind of being deemed as ‘you’re not horsey,’ ‘you don’t know what you’re talking about, just shut up’, do you know what I mean? … I think it’s got to be the people in the industry that actually need to start screaming a little bit more about things”.*

In other cases, participants were strongly opposed to public involvement in horse welfare in terms of training and management decisions. P5, in defence of tools and techniques in horse training that are often perceived negatively by the public, gave an example of maintaining alignment with existing equestrian culture:

*“Without education of like why and how we’re doing these things, many of the things that we do and love will fall to social licence and we have to kind of stop and say nope. Like that’s great that you want to have your say but you need to educate yourself before you have your say. And we have to stop the bureaucracies at the top and we have to stop it on the social licence. … It’s the equivalent of trying to parent your children by having a vote of all the school moms. Like this is a terrible idea. You don’t let everybody have a vote. You say no, this is what works for our parameters. I’ll respect what you do there, but I know that was best for my kid in this way, within reason. Like you still consult a dentist for your kid’s teeth and a doctor for their health and a teacher for their learning to read but we have to be careful not to let too much from the outside in because that I think could be far more dangerous”.*

Thus, while participants demonstrated concern about horse welfare, some also appear to be concerned with protection of their culture, and therefore their practices. Participants were generally unconvinced of the benefit that the public’s contribution might have on performance horse welfare, with some demonstrably opposed to public input. Indeed, public input on improving horse welfare appears to be unwanted and, at times, threatening to participants, which is also seen in other animal industry contexts (Heleski *et al.* 2006; Hubbard *et al.* 2007; Ventura *et al.* 2023). When individuals feel that their social group has been blamed for causing harms to others, they often react with resistance and justification strategies to protect their own sense of identity as well as that of their social group (Dersely & Wootton 2000; Gausel & Leach 2011; Barkan *et al.* 2012; Saguy *et al.* 2013).

#### Subtheme 3: Recognising harms and breaking away

While many participants perceived and are concerned about welfare issues in the performance horse industry, they often reframed, justified or trivialised their concerns. This is proposed as the result of dissonance between individuals’ concern for horses and the cultural context of equestrianism. However, not every participant appeared to use these potential dissonance reduction strategies. Rather, some described how they had broken away from enculturated practices and attitudes by tangibly prioritising horse welfare in their management, training, and performance activities. In these instances, participants spoke of being compelled by the *“normalised cruelties”* they described performance horses as often facing (P19). Such harms included *“horses living in isolation…not allowed to touch each other”* and riders that *“beat their horse quite badly”* whilst veterinarians, farriers, and other riders remained unconcerned (P18). P19 described how they left traditional equestrian culture because they believed that *“you could have a performance horse, and you could be successful and stand up for their well-being… the way I wanted to see them* [horses] *treated, and be in that* [performance] *world, I found it really, really hard to reconcile both of those things…so I left”.*

What motivates people to choose to leave situations in equestrianism for the sake of horse welfare likely varies between individuals. However, it could be that there may be a point where the dissonance between how horses are treated and the relationships with horses that individuals strive for cannot be resolved and that this discomfort leads to change. This may have been the case for P17, whose horse displayed problematic behaviours related to pain, but coaches, trainers and equestrian judges promoted traditional solutions such as: *“…a Pelham Segundo with really harsh points and tack nose bands and crank nose bands and just tightening, tightening, tightening. So all of the gadgets, all of the draw reins, everything to crank her face down…”.* P17 went on, *“I had all these trainers come up to me and say like, oh, you need to smack her when she pins her ears. You need to punch her in the face”.* After feeling that the mistreatment of performance horses had become normalised, this participant described how they had left competitive equestrian sport. They summarised the turning point in their thought process that began to lead to change: *“I was like, this horse has been nothing but kind to me. She’s been so sweet. I don’t want to keep hitting her”* (P17). Similarly, P8 shared a reflection about the process they went through in empathising with their horse’s perspective and choosing to set aside enculturated influences:

*“It’s when you’ve been taught from a young age that they* [horses] *have to do what you say, when you’re an adult going, I’m faced with this issue and everyone’s telling me that I have to get him to do it, but your brain says actually how would I feel about that? How would I feel? And then you go, hang on, let’s try something different”.*

While behaviour change is one possible form of dissonance resolution, as observed here, it is also least likely in situations where practices are rooted in strong attitudes (Festinger 1957; Cooper 2007; McGrath 2017), such as is likely the case in many equestrian sport circles. Self-reflection and critical evaluation of the emotions associated with a given practice then become essential in initiating change in practices, beliefs and attitudes (Mezirow 2000; Taylor 2000). In a study of youth sports and the influence of coaches (Fenoglio & Taylor 2014), the authors argue that those in sport who become aware of the difference between their own morals, ethics, and values and those of the subculture of their sport are likely susceptible to change. Similarly, an awareness of conflicts between one’s implicit attitudes and those held by a social group can also generate cognitive dissonance that creates openness to other perspectives (McConnell *et al.* 2008). These are important considerations in understanding how individuals might become receptive to change.

There is still much to be studied about equestrian culture. However, appreciating the role of enculturation in how equestrians are influenced to develop attitudes and behaviours that in some cases harm horses is critical. Contrasting potentially enculturated attitudes of equestrians towards horses and their welfare with the intrinsic attitudes of equestrians outside of the context of the performance horse industry would be useful. This could give insight into whether harmful attitudes are truly a product of enculturation or the result of other contributing factors. It could also be the case that enculturation is not always necessarily a negative influence. There may be ways that enculturation could promote positive change in the performance horse industry and this should be explored.

### General discussion

The present study provided rich data highlighting several human factors that may influence performance horse welfare. Study participants openly shared and reflected on horses and horse sports, a good life for horses, welfare issues, social norms in classical equestrian disciplines, and the public’s views of horse welfare in sports. From these discussions, five overarching themes were constructed, including perception of welfare issues, conflicting attitudes about the good life of a horse, objectification of the horse, instrumentalisation of care, and enculturation. A few recent studies have similarly investigated equestrian attitudes and perceptions using qualitative methods (Luke *et al.* 2023, 2024; Mauricio *et al.* 2024; Fiedler *et al.* 2025; Ross *et al.* 2025). The data presented here compliment previous works with a deeper contextualisation of findings in relation to theories of cognitive dissonance, enculturation, and care ethics.

*Theme 1* explored attitudes of participants and how they perceive and think about performance horse welfare. The results of the current research support other findings that equestrians in several disciplines are aware of welfare issues (Voigt *et al.* 2016; Horseman *et al.* 2017; Dubois *et al.* 2018a,b; Luke *et al.* 2023; Mauricio *et al.* 2024; Ross *et al.* 2025). Our participants discussed several concerning topics including social isolation, lack of access to turnout, and physical ‘beatings’. These admissions were revealing in that participants noted that they seem to go unreported or unresponded to at the time and so become normalised. This could be another aspect of the social group of enculturated participants, which reduces the likelihood of reporting abuses as seen in non-equestrian sports as well (Mountjoy *et al.* 2016). Nonetheless, discussing welfare issues of concern to participants led to the key revelation of potential underlying cognitive dissonance reduction strategies, such as justification, trivialisation, and reframing of welfare-compromising practices. In their study of equestrian knowledge, attitudes and beliefs about horse welfare, Mauricio *et al*. (2024) proposed that the dissonance between a love for horses and harmful management practices was due to lack of knowledge of what horses truly need for good welfare. However, in *Theme 2* of the present study, respondents largely conceptualised a good life for horses as being one where they were able *“to be a horse”.* Indeed, most descriptions of what this meant aligned with the Five Domains of welfare as defined by Mellor and colleagues (2020). This suggests that other barriers exist to the implementation of change beyond knowledge. One such barrier might be the conflict that participants seemed to be caught in when considering how the horse’s job contributes to living a good life whilst restricting the ability to *be a horse.*

Objectification of the horse was the focus of *Theme 3*, which gave insight into the relationship between humans and horses in the context of equestrian sports. While Nussbaum’s (1995) discussion of objectification was centred around feminist contexts, others have conceptualised objectification within other human relationships (Lacroix & Pratto 2015), including human relationships with animals. Indeed, several authors have proposed that animals raised for human food are objectified, leading to potential disregard for animal welfare (Cudworth 2008; Cole 2011; Morgan & Cole 2011; Leitsberger *et al.* 2016; Bos *et al.* 2018). Although to our knowledge the objectification of horses and effects on welfare have been under-described in the scientific literature, objectification is acknowledged in contemporary anthropology literature on horse-human relationships (Spannring 2016; Spannring & De Giorgio-Schoorl 2020; Vaught & Argent 2022). The objectification of performance horses may help to explain how harmful practices and attitudes have formed if they serve to maintain the usefulness of the horse. Taking a perspective from care ethics, we can comprehend how objectification is fundamentally detrimental to care since it distances the caregiver from realising the subjective needs of the one in care (Ricoeur 1986; Schaafsma 2014). Objectification of performance horses is perhaps not surprising given that their primary purpose is use in equestrian sport rather than as companions. Nonetheless, this is a potential barrier to improving equine welfare if the fundamental attitude towards horses is their objectification rather than one of value as a subjective being.

Such objectification of an individual may lead to the subsequent instrumentalisation of care (Engster 2006) as discussed in *Theme 4.* This has been reported in many contexts, including parental motivations for children’s education, healthcare campaigns, and religion (Fowers 2010; Sedgewick 2013; Lehrer *et al.* 2022. This may be occurring within equestrianism as well where horses may receive certain aspects of care for the primary purpose of achieving a performance outcome. An investigation by Pickering and Hockenhull (2019) found 85.7% of their survey participants wanted to know the instrumental benefits of following certain horse care advice in order to consider it valid. Combined with the findings in the present study, it appears that ensuring instrumental outcomes may be critical to equestrian decision-making when it comes to management practices.

Instrumentalisation of care in the context of non-human animals is seen as a moral violation by some welfarists and proponents of care ethics as applied to relationships between humans and non-human animals (Cooke 2021; Benz-Schwarzburg & Wrage 2023). Although instrumentalised care practices may often provide benefit to horses, it is argued that intent focused on instrumental outcomes reduces care *about* the individual (Tronto 1993) and morally harms the relationship between human and animal even in cases where welfare of the animal is considered to be good (Crary & Gruen 2022; Benz-Schwarzburg & Wrage 2023). Similarly, social licence may also be an example of instrumentalisation of care on a broader scale as it is used as one of the primary motivators in the equestrian industry to promote horse welfare interventions (Waran & Visser 2022). Thus, caring *for* horses in order to maintain social licence may instrumentalise this care when used for the purpose of furthering human goals.

In *Theme 5*, we examined enculturation in equestrianism. To the authors’ knowledge, this is the first study to explicitly address the issue of enculturation in equestrian sport and the potential role it plays regarding the persistence of practices and attitudes that compromise performance horse welfare. This theme not only demonstrates that enculturation in equestrian sports occurs, but how it can be problematic to horse welfare due to certain traditional attitudes common in equestrianism (Robinson 1999; Waran *et al.* 2007; van Weeren 2008; Thompson & Clarkson 2019). Pickering and Hockenhull (2019) investigated the preferred information sources for equestrians and found that they tended to be from other equestrians. This was also seen in the present study where participants expressed the need for other equestrians to be the sources of change within the culture. Enculturation may explain this biased sourcing of information and resistance to ‘outsider’ influence that seems present.

Particularly important within this theme is the reflection of a few respondents on their process of changing their practices and attitudes. In particular, these individuals recognised horses as sentient beings that experienced pain and suffering. According to Fiedler *et al.* (2025), this recognition of sentience may be critical to promoting updated horse welfare standards. Furthermore, the participants who shared their process of attitude and behaviour change expressed empathy for the horse when faced with welfare-compromising practices. Empathy for others is an important feature of caring *about* others as described by the care ethics framework (Held 1995; Noddings 2013).

More broadly, the conflicting attitudes towards horses and perceptions of welfare issues in terms of what participants believe a good life for horses consists of is a likely source of dissonance for equestrians. This is particularly in evidence with the contrast between *Themes 1* and *2.* Contradictions in attitudes that are demonstrated openly with those held internally have been shown to cause cognitive dissonance and thereby motivate dissonance-reduction strategies (Rydell *et al.* 2008). Motivation to reduce this dissonance may be related to the objectification of horses and instrumentalisation of care in that these could be used as dissonance-reduction strategies. Interestingly, objectification leads to resolution of cognitive dissonance by reducing the moral status of the animal, thereby making instrumentalisation and compromises in welfare morally acceptable to individuals (Joy 2009). It is possible that our participants used objectification of horses as a dissonance-reducing strategy to ease their cognitive discomfort. This could stem from an underlying personal ethical position (Galvin & Herzog 1992). Similarly, blocking outside influences and information sources is another dissonance-reduction strategy that may present a barrier to engaging equestrians to promote change as seen in *Theme 5.* Ultimately, we suggest that insight into the extent of cognitive dissonance experienced by equestrians may allow for identification of individuals who might be open to behaviour change intervention and help guide the development of effective strategies.

### Study considerations and future directions

This was an exploratory qualitative study meant to provide a foundation for future studies and not intended to be generalised across broader equestrian populations. However, the rich data provided allow the potential for transferability of the findings and analysis to other contexts (Braun & Clark 2022). Additionally, there were some important points that warrant further investigation. For example, digging deeper into the process of enculturation in equestrianism would be useful to understand equestrian culture. Exploring the relationship between humans and performance horses from a care ethics lens has similarly, to our knowledge, not yet been undertaken; such an approach may reveal more information regarding how equestrians care *about* horses. Further research focused on cognitive dissonance in equestrianism as it relates to horse welfare is also needed to develop effective dissonance-reduction strategies that also benefit horse welfare.

## Animal welfare implications and Conclusion

This study deepens our understanding of why horse welfare issues may persist in classical equestrian disciplines, by extending the frame of fundamental concern to socio-cultural factors. It is proposed here that enculturation and resultant attitudes that promote objectification of horses and the instrumentalisation of care are important to consider as potential root causes of performance horse welfare issues. As a result of perception of welfare issues and the internally conflicting attitudes likely held by equestrians involved in horse sports, motivation to reduce cognitive dissonance, through strategies such as those discussed in this study, also appear to be a potential barrier to change. It is hoped that these findings are useful in informing human behaviour change strategies to promote improved performance horse welfare.

## Supporting information

Cheung et al. supplementary material Cheung et al. supplementary material

## Supplementary material

The supplementary material for this article can be found at http://doi.org/10.1017/awf.2025.10028.
