# Supplementary material for: “But my horse is well cared for”: A qualitative exploration of cognitive dissonance and enculturation in equestrian attitudes toward performance horses and their welfare
Source: Anim Welf. 2025 Jul 24;34:e50. doi: 10.1017/awf.2025.10028 (PMC12304784; doi:10.1017/awf.2025.10028)
Supplement: Cheung et al. supplementary material [file S0962728625100286sup001.pdf]

“But my horse is well cared for”: A qualitative exploration of cognitive dissonance and enculturation in equestrian attitudes toward performance horses and their welfare

Erica Cheung https://orcid.org/0009-0007-3351-7910, Daniel Mills https://orcid.org/0000-0002-4765-9625 and Beth Ventura https://orcid.org/0000-0001-9476-6901

Animal Behaviour, Cognition, and Welfare Group, Department of Life Sciences, Joseph Banks Building, Green Lane, Lincoln LN6 7TS, UK

Author for correspondence: Erica Cheung, email: 28113818@students.lincoln.ac.uk

Supplementary material: Interview Guide Protocol

Interview Question 1:

1. Tell me about your horse(s)/ the horses you work with?
   a. What got you into having/caring for horses?
   b. What do you love about horses?
   c. How would you describe your personal relationship with horses you have owned or worked closely with?
   d. What are the main traits in horses that you admire?
   e. What do you consider to be the biggest influences on your attitudes toward horses?

Interview Question 2:

2. What sport/discipline are you involved in?
   a. How long have you been in it?

b. How did you get involved?
c. What, if anything, do you love most about [your sport]?
d. What keeps you wanting to stay in [the sport]?

<u>Interview Question 3:</u>

3. Changing topics a bit, I'm interested in your perspective- In your view, what does living a **good** life look like for horses?

a. In your experience, would you say that most horses in [your sport] meet those criteria/attributes/characteristics you just described? (why/why not)
b. How about with regard to training methods ?
   i. what does good training look like?
   ii. How do you think horses feel about being trained this way?
c. How about during the actual event?
   i. How do you think the horses experience the event?

<u>Interview Question 4:</u>

4. What (if any) **[insert language used by interviewee**] issues have you directly observed in [your sport]?

a. Are there other important issues that you're aware of occurring?
b. What do you think should be the top [**insert welfare language used by interviewee]** three priorities for [your sport]?
c. Do you think these <u>are</u> also the top 3 priorities for your sport, if not then what do you think their top 3 priorities are
d. Then for each priority (up to 6 issues): to what extent do you feel your sport is (or is not) addressing each of these issues?
   i. If yes: How?
   ii. If no: Why not?

<u>Interview Question 5:</u>

5. What other disciplines are you familiar with? What (if any) issues have you observed in that discipline? [Participant will be asked to choose one discipline]

a) Are there other important issues that you're aware of occurring?
b) What do you think should be the top [**insert welfare language used by interviewee]** three priorities for [the sport]?
c) Do you think these are also the top 3 priorities for the sport, if not then what do you think their top 3 priorities are?
d) Then for each priority (up to 6 issues): to what extent do you feel the sport is (or is not) addressing each of these issues?
   i. If yes: How?
   ii. If no: Why not?

Interview Question 6:

6. If you could change anything about how horses are cared for/managed in **your sport**, what would it be?
   a. Regarding day to day care/management of horses
   b. Regarding training methods
   c. Regarding the performance activities
   d. How about regarding your own horse(s)/horse(s) in your care?

Interview Question 7:

7. How do you think your sport (in terms of the management, training, and event) is seen by others?
   a. Family and friends
   b. Fellow sport community
   c. wider/ general horse community
   d. wider non-horse society

Interview Question 8:

8. Finally, [if you haven't covered it] what do you think are the public's main concerns about horse welfare in sports?
   a. To what extent do you feel these concerns are legitimate?
   b. What do you wish the public appreciated more about the welfare of horses within your discipline, if anything? (how horses are cared for and trained)

Demographic questions:

-If you feel comfortable to share, what gender do you identify as?
-If you feel comfortable to share, what age category do you belong in?

1. 18-24
2. 24-44
3. 44-60
4. 60-75
5. 75-90

Debrief:

-Thank the participant for their contribution and time
- Personal demographics are anonymous and only used to ensure population diversity for the study
-Remind participants that their data will be transcribed, anonymised, and analysed
-The transcribed, anonymised data may be quoted in the final thesis and publication(s)
-Their data may be used for the development of future research
-They can withdraw from the study up until data analysis
-Ask if participant would like to be acknowledged by name for their contributions or be acknowledged anonymously
-Ask if participant would like to be provided with the results of the study upon completion
-Final thank you
